# An AI-based algorithm for the automatic evaluation of image quality in canine thoracic radiographs

**DOI:** 10.1038/s41598-023-44089-4

**Published:** 2023-10-09

**Authors:** Tommaso Banzato, Marek Wodzinski, Silvia Burti, Eleonora Vettore, Henning Muller, Alessandro Zotti

**Affiliations:** 1 Department of Animal Medicine, Production and Health, University of Padua, Viale dell’Università 16, 35020 Legnaro, Padua Italy; 2 Department of Measurement and Electronics, AGH University of Krakow, PL32059 Krakow, Poland; 3 Information Systems Institute, University of Applied Sciences - Western Switzerland (HES-SO Valais), 3960 Sierre, Switzerland

**Keywords:** Radiography, Scientific data

## Abstract

The aim of this study was to develop and test an artificial intelligence (AI)-based algorithm for detecting common technical errors in canine thoracic radiography. The algorithm was trained using a database of thoracic radiographs from three veterinary clinics in Italy, which were evaluated for image quality by three experienced veterinary diagnostic imagers. The algorithm was designed to classify the images as correct or having one or more of the following errors: rotation, underexposure, overexposure, incorrect limb positioning, incorrect neck positioning, blurriness, cut-off, or the presence of foreign objects, or medical devices. The algorithm was able to correctly identify errors in thoracic radiographs with an overall accuracy of 81.5% in latero-lateral and 75.7% in sagittal images. The most accurately identified errors were limb mispositioning and underexposure both in latero-lateral and sagittal images. The accuracy of the developed model in the classification of technically correct radiographs was fair in latero-lateral and good in sagittal images. The authors conclude that their AI-based algorithm is a promising tool for improving the accuracy of radiographic interpretation by identifying technical errors in canine thoracic radiographs.

## Introduction

Radiography is the most widely used imaging technique for the evaluation of the canine thorax^1^. Obtaining high-quality images is essential for correct radiographic interpretation, and overlooking proper technique can lead to misinterpretation of several radiographic signs^2^. The topic of radiographic image quality has been scarcely investigated in veterinary medicine, with only a few papers available on the subject^3,4^. Additionally, the incidence and causes of radiographic technical errors in veterinary clinical practice are poorly understood^5^. In human medicine, specific guidelines outlining acceptable diagnostic image quality standards are available^6^. However, to the best of the authors' knowledge, such guidelines do not exist in veterinary medicine.

The use of artificial intelligence (AI) in veterinary diagnostic imaging is experiencing a rapid increase in popularity, as more veterinarians become aware of the benefits offered by this technology^7^. This has led to a corresponding rise in the number of published works exploring the various applications of AI in the field of veterinary medicine. Particularly in the last few years, studies on the applications of AI in classifying canine meningiomas from MR^8^, in distinguishing between meningiomas and gliomas in MR^9^, and in detecting spinal cord diseases from MR images^10^ have been published. To date, the most prolific sector of investigation in this field is the application of AI for the automatic detection of lesions from thoracic x-rays with an increasing number of publications on this topic^11–14^.

Recent years have seen a growing interest in the use of AI for the automatic evaluation of the quality of medical images and in human medicine, and several AI-based algorithms have been developed for the quality evaluation of chest X-ray images, with promising results^15,16^. However, to the best of the authors’ knowledge, such tools are as yet unavailable in veterinary medicine. Thus, the aim of this study was to develop and test an AI-based algorithm for the automatic evaluation of the quality of chest radiographs in veterinary medicine.

## Materials and methods

### Database creation

The archives of three different veterinary clinics - namely the Veterinary Teaching Hospital of the University of Padua (Legnaro, Padova, Italy), the Pedrani Veterinary Clinic (Zuliano, Vicenza, Italy) and the Strada Ovest Veterinary Clinic (Treviso, Italy) were used in this project. Three different X-ray systems were used (1- FDR D-EVO 1200 G43 (Fujifilm Corporation) digital radiology (DR) at the Veterinary Teaching Hospital of the University of Padua, 2- a Isomedic RT 800 MA (Isomedic S. r. L) at the Pedrani Veterinary Clinic, 3- FCR PRIMA T2 (Fujifilm Corporation) at the Strada Ovest Veterinary Clinic). Canine thoracic radiographs, acquired in latero-lateral (both left and right) and in sagittal (both ventro-dorsal and dorso-ventral) projections were collected from the databases of the three institutions.

### Image analysis

The images were assessed simultaneously by three of the authors (TB, SB, and EV, with 13, 5 and 1 years of experience in veterinary diagnostic imaging respectively) in a Digital Imaging and Communication in Medicine (DICOM) format using a freely available image visualization and analysis software (Horos, Nimble). The tags were assigned following a consensus discussion. The tags used for the evaluation of image quality were: (a) correct, (b) rotated (rotation was evaluated by checking for superimposition of opposite ribs in latero-lateral images, and of the sternum and vertebral column in sagittal images), (c) underexposed (an image was classified as underexposed if quantum mottle was evident or if the pulmonary structures were not clearly evident due to an overall lack of detail), (d) overexposed (when some portions of the image were completely black), (e) limbs (if the limbs were incorrectly positioned), (f) neck (if the neck was too flexed or too extended), (g) blurred (if motion artifacts were seen, with evident distortion of the anatomical structures), (h) cut (if a portion of the thorax was excluded from the radiograph), (i) foreign object (if any examples of these, or medical devices, were present). All the tags, except for “correct”, were not mutually exclusive and therefore a multi-label deep-learning approach was used. The evaluation of exposure is, to a certain extent, subjective and, therefore, to make the evaluation more objective, a radiograph was rated as underexposed if quantum mottle was evident within the entire image, especially affecting the bony trabecular pattern^17^. On the other hand, a radiograph was rated as overexposed if only some areas of the radiograph remained completely black despite changing brightness and contrast. The position of the limbs was rated as incorrect if a superimposition of the limbs on the thoracic structures was evident. The position of the neck was rated as incorrect in the case of abnormalities in the position of the trachea (over-extension or over-flexion) in latero-lateral radiographs. Neck mispositioning was not considered in sagittal radiographs.

### Deep learning

The DICOM files were initially converted to the MetaImage Medical Format (MHA) format, resampled to 224 × 224 pixels and normalized by a Z-normalization specific to the ResNet-50 network. The ResNet-50 pre-trained on ImageNet was used, since previous research has indicated that it provides the most accurate results for X-ray classification with a limited size datasets^11–14^. The architecture was then fine-tuned on the aforementioned database with a multi-label setting, as the quality classes were not mutually exclusive. Binary cross-entropy was employed as the objective function, the Adam algorithm as the stochastic optimizer, and an exponential scheduler was used to reduce the learning rate after each epoch. The images set was randomly split into a training, validation and test set comprising 80%, 10% and 10% of the images respectively. The training set was augmented online through standard transformations, including affine transformation, random cropping, flips, and contrast changes. The training was conducted on a workstation (Linux operating system; Ubuntu 18.04, Canonical) devoted to deep learning, equipped with four GPUs (4x Tesla V100; NVIDIA and Canonical), a 2.2 GHz processor (Intel Xeon E5-2698 v4; Intel) and 256 GB random access memory. The evaluation metrics were not directly optimized or utilized during training, nor was the metadata related to the source institution deployed to guide the training process.

### Statistical analysis

All the statistical analyses were performed using a custom-built Python programming language script (Python Software Foundation; the Python Language Reference, version 3.6; available at http://www.python.org). The performance of ResNet-50 was evaluated by means of the receiver-operator characteristics curve (ROC) and the area under the receiver-operator characteristics curve (AUC); the sensitivity, the specificity, and the positive and negative likelihood ratios (PLR and NLR, respectively), along with their 95% confidence intervals, were also calculated. The performance of ResNet-50 for each quality parameter was rated as excellent (AUC ≥ 0.9) high (0.9<AUC ≥ 0.8) fair (0.8 < AUC ≥ 0.7), or poor (AUC < 0.7)^17^. All *P-*values were assessed at an alpha of 0.05.

### Ethics approval

This study was conducted respecting the Italian law 26/2014 (that transposes the EU directive 2010/63/EU). As the data used in this study were part of routine clinical activity, no ethical committee approval was required. Informed consent regarding personal data processing was obtained from the owners.

## Results

### Database

**Figure 1:**
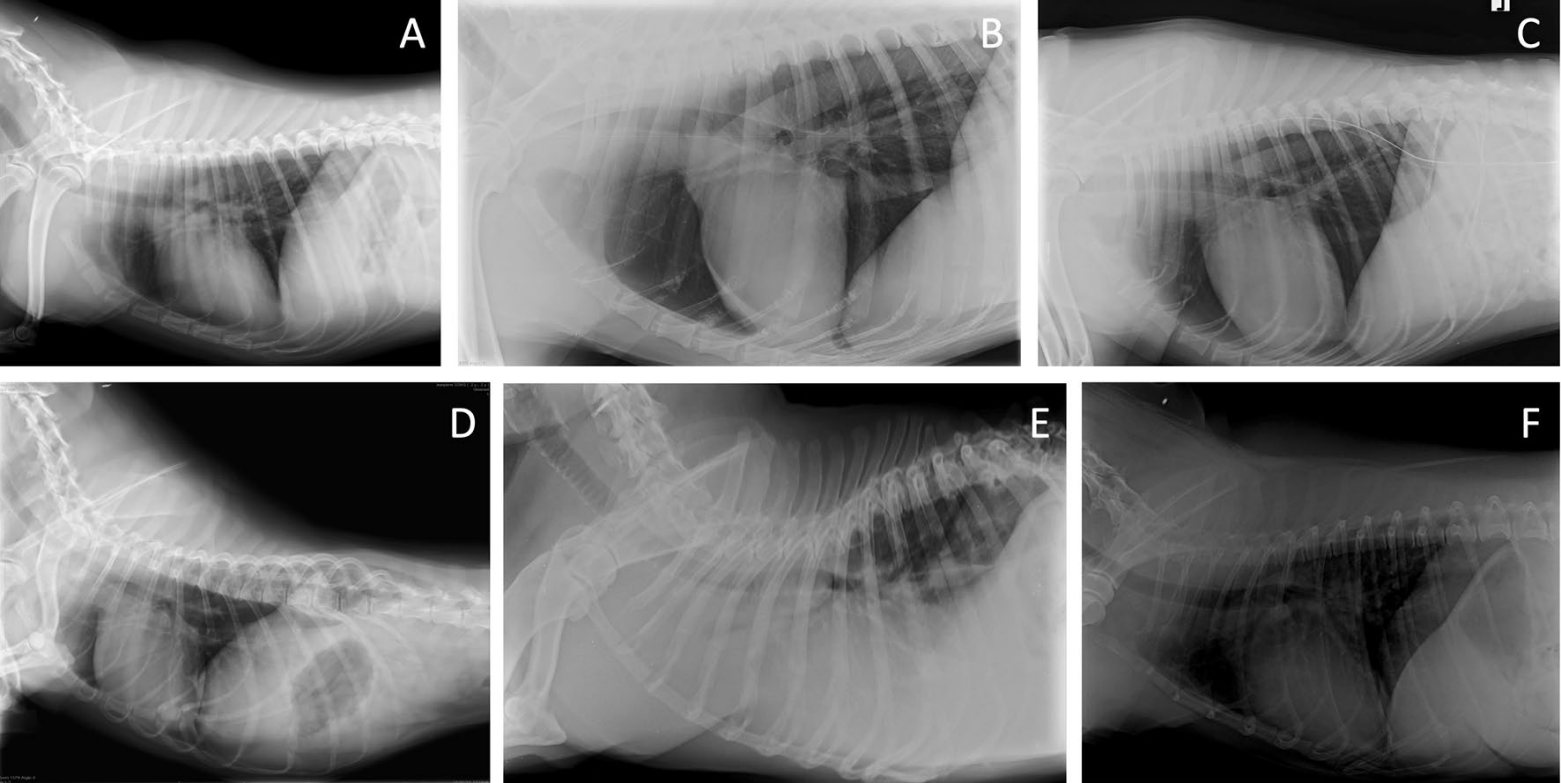
Example images of latero-lateral radiographs showing the quality issues included in the study. (**A**) Correct, (**B**) Neck mispositioning, (**C**) Foreign object, (**D**) Rotated, neck mispositioning, limb mispositioning, (**E**) Underexposed, (**F**) Overexposed.

**Figure 2:**
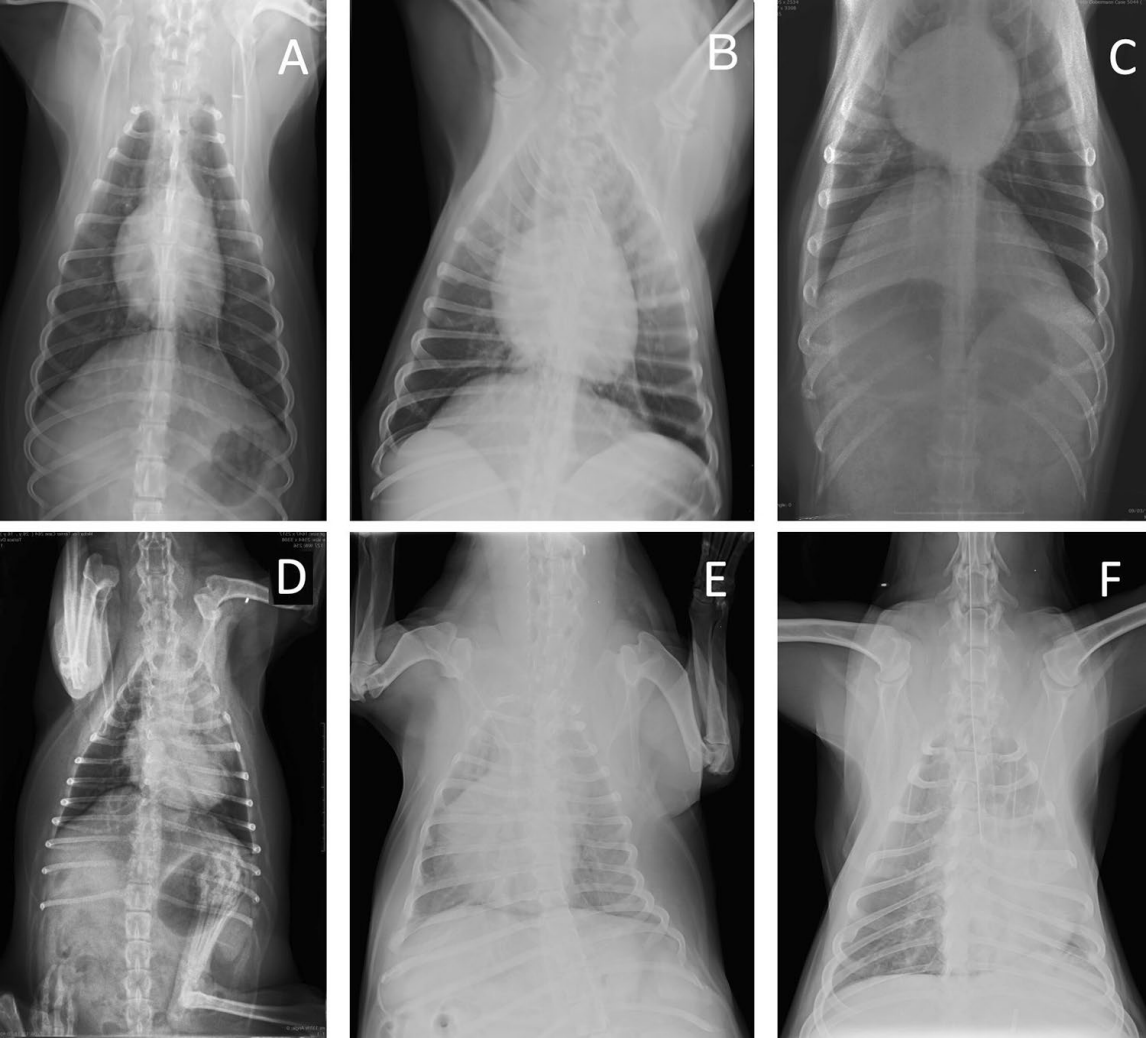
Example images of sagittal radiographs showing the quality issues included in the study. (**A**) Correct, (**B**) Blurred, (**C**) Cut, (**D**) Rotated, limb mispositioning, (**E**) Underexposed, rotated, (**F**) Foreign object, underexposed.

Overall 6028 latero-lateral and 4053 sagittal radiographs were included in the database. Left and right latero-lateral projections were grouped together. In the same way, ventro-dorsal and dorso-ventral (sagittal) radiographs were also grouped together. The number of radiographs for each tag are listed in Tables 1, 2. As multiple quality issues were present in several radiographs, the total number of tags exceeded the total number of radiographs. 1252 latero-lateral and 854 sagittal radiographs were discarded as belonging to skeletally immature dogs. All the included radiographic tags were included in the training, validation and test sets. Example images of some of the included tags for latero-lateral and sagittal radiographs are reported in Figs. 1 and 2 respectively.

**Table 1:** Summary of the radiographic abnormalities detected on the training, validation and test sets of the latero-lateral radiographs.

Radiographic finding	Number of radiographs		
	Training	Validation	Test
Correct	3517	458	487
Blurred	43	7	5
Cut	135	8	10
Foreign object	161	22	19
Limb mispositioning	116	18	17
Underexposed	189	21	18
Overexposed	157	18	20
Rotated	703	56	81
Neck mispositioning	112	11	13

**Table 2:** Summary of the radiographic abnormalities detected on the training, validation and test sets of the sagittal radiographs.

Radiographic finding	Number of radiographs		
	Training	Validation	Test
Correct	1949	389	247
Cut	114	22	22
Foreign object	62	6	6
Limb mispositioning	37	4	8
Underexposed	169	33	29
Overexposed	57	12	8
Rotated	757	146	115

### Classification results

The complete classification results for the radiographic quality indices are reported in Tables 3, 4. Applying the proposed AI-based tool on the latero-lateral radiographs resulted in variable performances for the different quality indices: in fact, it had an excellent accuracy only for limb mispositioning and a high accuracy for blurriness, foreign object, underexposure, overexposure, rotation and neck mispositioning. The accuracy in classifying normal radiographs was only fair. The overall accuracy was 81.5 %.

**Table 3:** Performance of ResNet.50 in the test set of the latero-lateral radiographs.

Radiographic finding	AUC	Sensitivity	Specificity	PLR	NLR
Correct	0.77 (0.72–0.81)	0.6 (0.56–0.64)	0.78 (0.71–0.85)	2.8 (2–3.8)	0.5 (0.45–0.56)
Blurred	0.83 (0.66–0.92)	0.6 (0.17–0.92)	0.92 (0.89–0.94)	7.8 (3.66–16.9)	0.43 (0.14–1.26)
Cut	0.84 (0.71–0.97)	0.8 (0.44–0.96)	0.67 (0.63–0.7)	2.4 (1.72–3.3)	0.2 (0.08–1)
Foreign object	0.81 (0.66–0.93)	0.63 (0.38–0.82)	0.90 (0.88–0.92)	6.8 (4.47–10.42)	0.4 (0.22–0.73)
Limb mispositioning	0.93 (0.87–0.98)	0.82 (0.56–0.95)	0.89 (0.86–0.91)	7.82 (5.69–10.76)	0.20 (0.07–0.55)
Underexposed	0.88 (0.79–0.94)	0.83 (0.57–0.95)	0.83 (0.79–0.86)	4.8 (3.7–6.2)	0.2 (0.07–0.57)
Overexposed	0.87 (0.8–0.95)	0.85 (0.61–0.96)	0.76 (0.72–0.79)	3.5 (2.8–4.4)	0.19 (0.06–0.56)
Rotated	0.84 (0.80–0.88)	0.76 (0.65–0.84)	0.7 (0.66–0.73)	2.56 (2.1–3)	0.33 (0.22–0.5)
Neck mispositioning	0.87 (0.73–0.99)	0.92 (0.8–1)	0.78 (0.56–0.84)	5 (3.3–6.9)	0.3 (0.12–0.65)

Values in parenthesis are 95% confidence intervals.

**Table 4:** Performance of ResNet 50 on the test set of the sagittal radiographs.

Radiographic finding	AUC	Sensitivity	Specificity	PLR	NLR
Correct	0.81 (0.77–0.86)	0.78 (0.72–0.83)	0.68 (0.6–0.75)	2.41 (1.88–3.1)	0.32 (0.25–0.42)
Cut	0.86 (0.69–0.97)	0.66 (0.3–0.92)	0.8 (0.76–0.84)	3.4 (2–5.6)	0.4 (0.16–1)
Foreign object	0.8 (0.63–0.94)	0.7 (0.3–0.96)	0.71 (0.66–0.75)	2.46 (1.5–4)	0.4 (0.12–1.3)
Limb mispositioning	0.88 (0.7–0.96)	0.71 (0.3–0.96)	0.8 (0.75–0.84)	3.52 (2.12–5.9)	0.36 (0.11–1.2)
Underexposed	0.92 (0.76–1)	0.83 (0.59–0.97)	0.81 (0.76–0.85)	4.32 (3.22–5.79)	0.21 (0.07–0.58)
Overexposed	0.84 (0.67–0.93)	0.66 (0.09–99)	0.64 (0.6–0.7)	1.88 (0.83–4.2)	0.52 (0.1–2.57)
Rotated	0.84 (0.67–0.98)	0.77 (0.66–0.85)	0.81 (0.77–0.86)	4.13 (3.17–5.37)	0.29 (0.2–0.42)

Values in parenthesis are 95% confidence intervals.

On the sagittal radiographs, only 8 images were classified as blurred and therefore this latter quality index was not included in the model. The performance of the proposed AI tool on sagittal radiographs was high for all the considered quality indices except for underexposure, which was excellent (AUC = 0.92). The overall accuracy was 75.7%.

## Discussion

The present study suggests that deep learning may be a valuable tool for automatically evaluating the quality of both sagittal and latero-lateral canine thoracic radiographs. This option would be highly beneficial in situations where an expert veterinary radiologist is not readily available, such as when centres rely on external consultation services or when an expert radiologist is only occasionally present. Overall, the ability to automatically evaluate image quality has the potential to improve efficiency and effectiveness in the veterinary medical imaging field.

In this prospective quality-improvement study, the quality criteria for chest radiographs were derived from the indications given in textbooks^2^, while also incorporating elements from prior works on the automatic evaluation of chest radiographs in human medicine^15,16^. Radiographic abnormalities were evaluated by the authors based on their expertise in veterinary diagnostic imaging, which thus involved some degree of subjectivity. In order to, at least partially, overcome this subjectivity, the radiographs were evaluated simultaneously by three different experienced operators.

Not surprisingly, one of most common quality issue encountered on our database was a lack of parallel (in 840 latero-lateral radiographs) and perpendicularity (in 1018 sagittal radiographs) between the animal and the detector, labelled as “rotated” in this paper. This quality index is also frequently reported in human medicine, with Nousiainen et al.^16^ proposing an automated methodology for chest radiograph quality control using convolutional neural networks (CNNs). Rotation was evaluated subjectively during that study, and the deep learning-based approach had an AUC of 0.72 for detecting a quality issue of that type. Instead, the model presented here, demonstrated a higher accuracy (AUC of 0.84) for rotation, likely due to the larger size of our training database. Another study, by Meng et al.^15^, also examined the automatic evaluation of human chest X-rays, including the assessment of rotation. However, it is difficult to directly compare the results of our study with those of Meng et al.^15^ as the methods used were quite different; in fact, Meng et al.^15^ developed a complex method to automatically measure the degree of rotation. However, the accuracy of this latter method for detecting rotation was limited.

In the present study, the accuracy for classifying both underexposed and overexposed radiographs was high, with AUCs between 0.84 and 0.92 in the different datasets. This result was rather unexpected because the radiographs included in the study were obtained using both computed radiology (CR) devices and direct radiology (DR) systems. It is known that underexposure appears slightly differently in CR than in DR^16^. Nonetheless, the high accuracy achieved in this study suggests that the developed algorithm was able to identify common features of underexposure in both modalities. To the best of our knowledge, this is the first study proposing a deep learning-based algorithm to evaluate such quality indices and, therefore, a comparison with similar studies is not possible.

The presence of any foreign object on the radiograph was recorded and included in the quality indices. While these foreign objects are not a quality issue in and for themselves, they can sometimes obscure important areas of the image, making it difficult to detect certain lesions. Most of the time, these objects are medical devices that are vital to the patient (e.g. metallic clips, tracheal or oesophageal tubes, chest drainages). To the best of our knowledge, the influence of foreign bodies on the accuracy of AI-powered diagnostic tools has not yet been investigated. However, it can be postulated that their presence might interfere with the interpretation of the images by the algorithms, as these objects are superimposed on thoracic structures.

Mispositioning of the limbs is a common issue in latero-lateral radiographs, and this can hinder interpretability due to the superimposition of the shoulder and forelimb muscles and bones on the cranial portion of the thorax, potentially obscuring lesions in that region^15^. The developed network had a high accuracy (AUC = 0.93 on latero-lateral, and AUC = 0.92 on sagittal) in detecting this technical error, suggesting that it was readily identified by ResNet-50. In our opinion, this quality index is less prone to subjectivity, and the evaluation by the three experienced radiologists may have been more consistent, leading to the high accuracy of the network.

One limitation of this study is that the respiration phase was not considered among the quality indices. Other similar studies in human medicine have included this quality index in their analysis^16^. We elected not to include inspiration among the quality indices because there are no objective criteria for evaluating the appropriateness of the respiratory phase in the literature, and such an assessment would therefore be very subjective and prone to high inter- and intra-rater variability.

The overall accuracy of the generated system exhibited a slightly superior performance on latero-lateral images (total accuracy 81.5%) than on sagittal images (total accuracy 75.5%). It is the authors’ opinion that this discrepancy is largely due to the smaller size of the sagittal image database in comparison to the latero-lateral radiograph database. Employing a more extensive database could potentially enable higher overall results to be achieved during classification.

## Conclusions

This study presents a deep learning-based algorithm for detecting common quality issues in sagittal and latero-lateral radiographs. The developed algorithm had high accuracy in detecting limb mispositioning, as well as high accuracy in detecting other issues such as blurred images, foreign objects, underexposure, overexposure, rotation, and neck mispositioning. The algorithm had fair accuracy in classifying normal radiographs.

## Data Availability

The datasets generated and/or analysed during the current study are not publicly available due to privacy restrictions but are available from the corresponding author on reasonable request.

## References

[1] Keyserling CL, Buriko Y, Lyons BM, Drobatz KJ, Fischetti AJ (2017). Evaluation of thoracic radiographs as a screening test for dogs and cats admitted to a tertiary-care veterinary hospital for noncardiopulmonary disease. Vet. Radiol. Ultrasound.

[2] Thrall DE (2018). Principles of Radiographic Interpretation of the Thorax. Textbook of Veterinary Diagnostic Radiology.

[3] Dixon J, Biggi M, Weller R (2018). Common artefacts and pitfalls in equine computed and digital radiography and how to avoid them. Equine Vet. Educ..

[4] Jackson MA (2011). Identification and prevalence of errors affecting the quality of radiographs submitted to Australian thoroughbred yearling sale repositories. Vet. Radiol. Ultrasound.

[5] Ewers RS, Hofmann-Parisot M (2000). Assessment of the quality of radiographs in 44 veterinary clinics in Great Britain. Vet. Rec..

[6] Blanc D (1998). European guidelines on quality criteria for diagnostic images. Radioprotection.

[7] Wilson DU, Bailey MQ, Craig J (2022). The role of artificial intelligence in clinical imaging and workflows. Vet. Radiol. Ultrasound.

[8] Banzato T, Cherubini GB, Atzori M, Zotti A (2018). Development of a deep convolutional neural network to predict grading of canine meningiomas from magnetic resonance images. Vet. J..

[9] Banzato T, Bernardini M, Cherubini GB, Zotti A (2018). A methodological approach for deep learning to distinguish between meningiomas and gliomas on canine MR-images. BMC Vet. Res..

[10] Biercher A (2021). Using deep learning to detect spinal cord diseases on thoracolumbar magnetic resonance images of dogs. Front. Vet. Sci..

[11] Banzato T (2021). Automatic classification of canine thoracic radiographs using deep learning. Sci. Rep..

[12] Burti S, Longhin Osti V, Zotti A, Banzato T (2020). Use of deep learning to detect cardiomegaly on thoracic radiographs in dogs. Vet. J..

[13] Boissady E, de La Comble A, Zhu X, Hespel AM (2020). Artificial intelligence evaluating primary thoracic lesions has an overall lower error rate compared to veterinarians or veterinarians in conjunction with the artificial intelligence. Vet. Radiol. Ultrasound.

[14] Adrien-Maxence H (2022). Comparison of error rates between four pretrained DenseNet convolutional neural network models and 13 board-certified veterinary radiologists when evaluating 15 labels of canine thoracic radiographs. Vet. Radiol. Ultrasound.

[15] Meng Y (2022). Automated quality assessment of chest radiographs based on deep learning and linear regression cascade algorithms. Eur. Radiol..

[16] Nousiainen K, Mäkelä T, Piilonen A, Peltonen JI (2021). Automating chest radiograph imaging quality control. Phys. Med..

[17] Jiménez DA, Armbrust LJ, O’Brien RT, Biller DS (2008). Artifacts in digital radiography. Vet. Radiol. Ultrasound.

